# Cell-cell fusion as a mechanism of DNA exchange in cancer

**DOI:** 10.18632/oncotarget.23715

**Published:** 2017-12-27

**Authors:** Stephen C. Searles, Endi K. Santosa, Jack D. Bui

**Affiliations:** ^1^ Department of Pathology, University of California, San Diego, La Jolla, CA, 92093, USA

**Keywords:** cell-cell fusion, cancer heterogeneity, chemoresistance, clonal diversity, aneuploidy

## Abstract

Cell-cell fusion describes the process by which two cells combine their plasma membranes and become a single cell, possessing and retaining certain genetic information from each parent cell. Here, using a Cre-*loxP*-based method initially developed to investigate extracellular vesicle targeting, we found that cancer cells spontaneously and rapidly deliver DNA to non-cancer cells *in vitro* via a cell-cell fusion event. The resulting hybrid cells were aneuploid and possessed enhanced clonal diversity and chemoresistance compared to non-hybrid cancer cells. We also observed cell-cell fusion to occur *in vivo* between melanoma cells and non-cancer cells of both hematopoietic and non-hematopoietic lineages. These findings suggest that cell-cell fusion occurs during the natural progression of cancer and show that this mechanism has the potential to cause massive genomic alterations that are observed in cancer. Furthermore, these findings somewhat contradict recent publications suggesting that the Cre-*loxP* method measures only extracellular vesicle-mediated intercellular communication.

## INTRODUCTION

Cell-cell fusion refers to the process by which two or more cells combine their plasma membranes to become a single hybrid cell containing DNA from each parent cell [1]. This fundamental biological process has been well documented in many organisms, including plants [2], yeast [3], *C. elegans* [4], *D. melanogaster* [5], and higher eukaryotes [6]. The functional consequence of cell-cell fusion is the formation of a hybrid cell that can maintain genotypic and phenotypic properties of both parent cells. In this sense, cell-cell fusion is a robust mediator of cellular reprogramming that can lead to the creation of cells with novel properties [7].

In the context of cancer, it has been hypothesized that cell-cell fusion may act to increase the genotypic and phenotypic diversity of daughter cells [8]. This mechanism of DNA exchange, via “sexual reproduction” (fusion and subsequent reductive division), is thought to be a more efficient way to generate populational heterogeneity as opposed to simply relying on the accumulation of oncogenic mutations in a single cell (“asexual reproduction”). Based on this hypothesis, hybrid cells are more likely to possess characteristics that would allow for the progressive growth of cancer compared to non-hybrid cells. These characteristics include rapid proliferation [9], cancer stem-cell formation [10], resistance to chemotherapeutics [11, 12], and metastasis [13, 14], among others. Fusion has been reported to occur in many types of cancer, including breast, melanoma, sarcoma, glioblastoma, renal cell carcinoma, and ovarian carcinoma [15, 16]. However, only few studies have quantified cell-cell fusion *in vivo* [17], and to our knowledge, none have clearly identified which non-cancer cells are capable of fusing with cancer cells *in vivo*. While definitive evidence linking cell fusion to cancer progression in humans is lacking, it has become increasingly clear using animal models that cell fusion plays a physiologically relevant role in the progression of cancer [18], especially as it relates to metastasis [19, 20], drug resistance [21], and cancer stem cell formation [9, 10].

Extracellular vesicles (ECVs) have recently been recognized as major mediators of intercellular communication in numerous physiological processes, including cancer [22, 23]. ECVs encompass both secreted exosomes as well as small vesicles that are shed directly from the plasma membrane [24]. The molecular contents of ECVs remain biologically active and can be transferred to cells locally and distally, resulting in the cellular reprogramming of targeted cells. ECV cargo includes proteins [25], lipids [26], and nucleic acids [27], all of which have been shown to possess biological activity. Recent publications have elegantly demonstrated the profound role of tumor-derived ECVs in modulating tumorigenic processes, including immune-evasion, angiogenesis [28], pre-metastatic niche formation [29], and metastatic organotropism [30]. In particular, it appears that tumor-derived ECVs are able to modulate and reprogram host cells to provide a more hospitable environment for cancer cells to grow. Despite this recent flood of new information regarding this process, the identity of which specific cells uptake ECVs in truly physiological conditions remains unresolved.

In this study we developed a Cre-*loxP* model system initially to investigate how molecular information is transferred out of cancer cells via ECVs. We unexpectedly found that cancer cells and non-cancer cells spontaneously and rapidly combine DNA via a fusion event that could affect cancer cell ploidy, heterogeneity, and fitness. These studies document and quantify cell-cell fusion *in vitro* and *in vivo* using transplantable murine tumor models and show that this process could serve as an engine to drive cancer aneuploidy and heterogeneity.

## RESULTS

### Cancer cells rapidly transfer Cre to fibroblasts and macrophages *in vitro*

**Figure 1:**
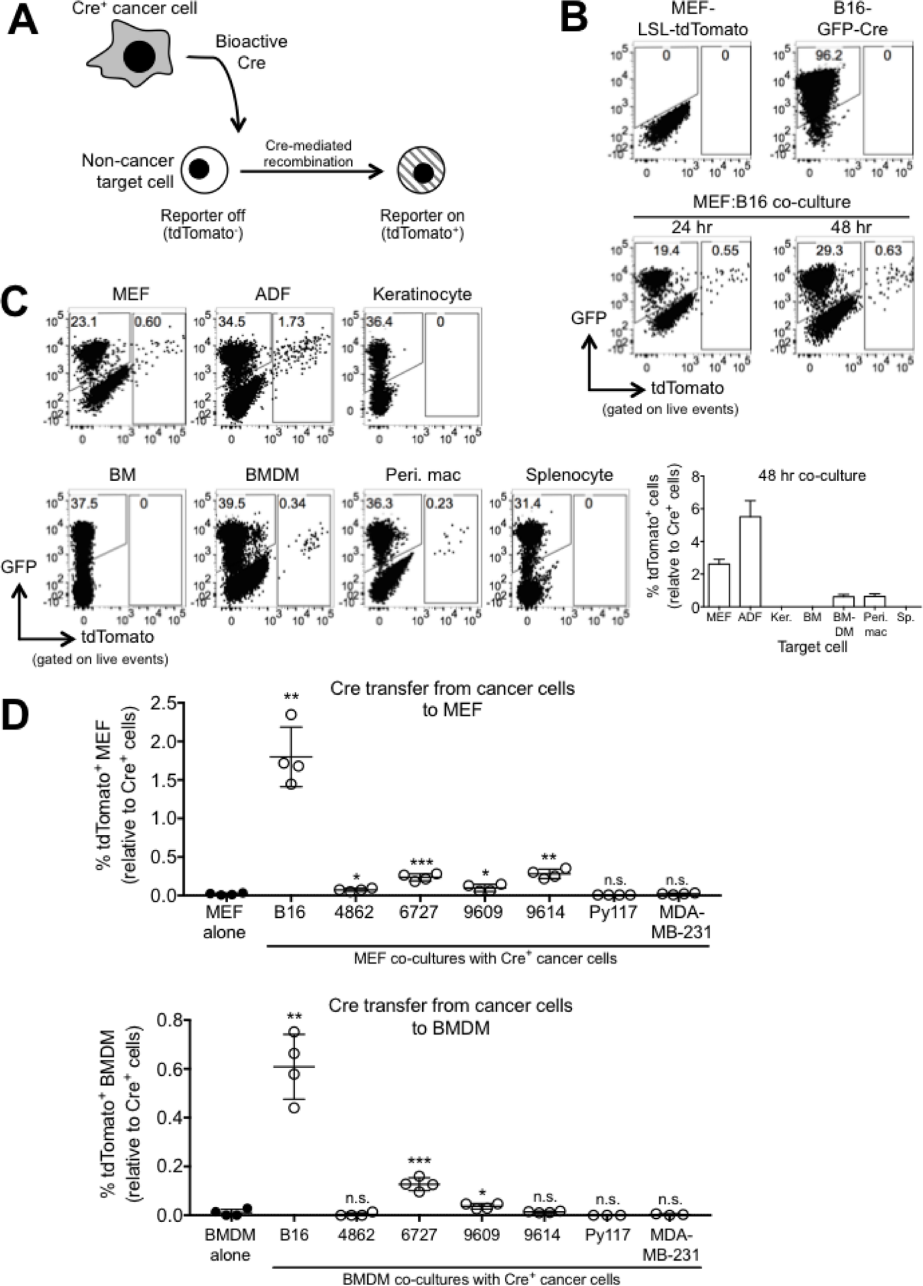
Bioactive Cre is rapidly transferred from cancer cells to non-cancer cells *in vitro* (**A**) Schematic representation depicting the Cre-*loxP* model system used to investigate the exchange of molecular information between cancer cells and non-cancer cells. (**B**) FACS plots showing GFP and tdTomato expression in reporter MEF (LSL-tdTomato), B16-GFP-Cre cells, and 24- and 48 hr B16:MEF co-cultures. (**C**) Representative FACS plots and quantification of tdTomato expression in 48 hr co-cultures of B16-GFP-Cre and different reporter cells including MEF, adult dermal fibroblasts (ADF), keratinocytes (Ker.), bone marrow (BM), BM-derived macrophages (BMDM), peritoneal macrophages (Peri. mac), and splenocytes (Sp.) (*n* = 3 or 4 independent experiments). The relative percentage of tdTomato^+^ cells is shown, and was calculated by dividing the frequency of tdTomato^+^ cells by the frequency of GFP-Cre^+^ cells in each co-culture. Data is represented as mean ± SEM. (**D**) Quantification of tdTomato expression in 48 hr co-cultures of various different GFP-Cre-expressing cancer cell lines (B16 melanoma, 4862, 6727, 9609, and 9614 MCA sarcoma, Py117 and MDA-MB-231 breast cancer) with reporter MEF or BMDM (*n* = 3 or 4 independent experiments). The relative percentage of tdTomato^+^ cells is shown, and was calculated by dividing the frequency of tdTomato^+^ cells by the frequency of GFP-Cre^+^ cells in each co-culture. Data is represented as mean ± SEM. Symbols represent statistically significant increases in tdTomato+ cells compared against reporter cells alone.

We initially sought to identify which healthy host cells are capable of receiving cancer-derived molecular information, and then to determine how this communication affects the behavior of these cells. To this end, we established a Cre-*loxP* system consisting of cancer cells that express Cre recombinase and non-cancer cells that contain a reporter locus consisting of a floxed stop codon preceding tdTomato (*loxP*-STOP-*loxP*-tdTomato, or LSL-tdTomato) under control of the ROSA promoter. In this model system, if a non-cancer cell receives cancer-derived Cre, the reporter will be activated and the non-cancer cell will turn red via expression of tdTomato (Figure 1A).

As an initial proof-of-concept that Cre transfer occurs between cancer and non-cancer cells, we co-cultured mouse embryonic fibroblasts (MEFs) derived from reporter mice (B6.Cg-Gt(ROSA)26Sortm9(CAG-tdTomato)Hze/J) with B16.F10 melanoma cells expressing GFP-Cre (B16-GFP-Cre) for 24 and 48 hours and then measured tdTomato fluorescence by FACS. We could detect tdTomato^+^ cells after 24 hours, indicating that Cre transfer occurred rapidly between B16 and reporter MEF cells *in vitro* (Figure 1B). The percentage of fused cells was 0.55% at 24 hours and 0.63% at 48 hours, indicating that the fusion occurred quickly and continued to occur. The apparent decrease in rate of fusion (0.08% between 24 and 48 hours) was likely due to the rapid proliferation of B16 tumor cells, which are included in the denominator of the calculation. B16-derived Cre was transferred to other cell types derived from reporter mice, including adult dermal fibroblasts (ADF), bone marrow-derived macrophages (BMDM), and peritoneal macrophages, albeit with differing levels of efficiency (0.5–5%) (Figure 1C). We expressed GFP-Cre in an expanded panel of cancer cell lines encompassing 4 MCA sarcomas (4862, 6727, 9609, 9614 [31]) and 2 breast cancer cells (MDA-MB-231 and Py117 [32]) and then co-cultured these cells with reporter MEF and BMDM for 48 hours. We found that some of the MCA sarcoma cell lines could induce low levels of reporter activation in target MEF and BMDM, but none were as efficient as transferring Cre to target cells than B16 cells. We also found that neither of the breast cancer cell lines tested could induce reporter activation in MEF or BMDM during the 48 hour co-culture period to any significance (Figure 1D). These data demonstrate that melanoma cells seem particularly well adept at transferring molecular information to non-cancer target cells in the conditions tested here, but also show that this phenomenon can occur with other types of cancer cells, albeit at lower efficiency.

### B16-GFP-Cre ECVs contain Cre RNA

**Figure 2:**
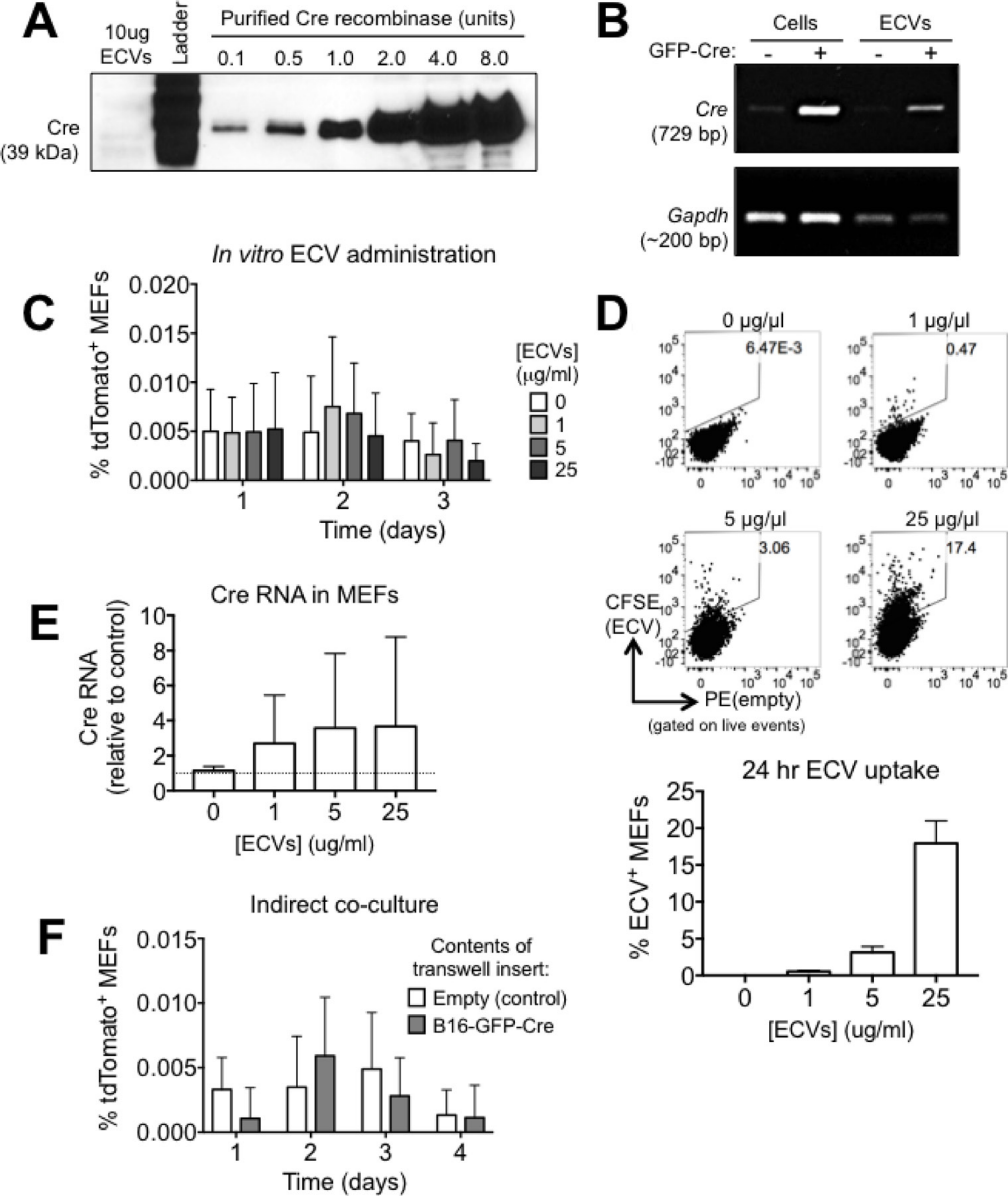
The rapid transfer of Cre from B16 melanoma cells to MEF is not mediated by ECVs (**A**) Analysis of Cre protein in 10 μg of B16-GFP-Cre ECVs by western blotting. (**B**) Analysis of Cre RNA in B16-GFP-Cre ECVs by PCR. (**C**) Quantification of the frequency of tdTomato^+^ MEF after treatment with B16-GFP-Cre ECVs for up to three days (*n* = 4 independent experiments). Data is represented as mean ± SEM. (**D**) Representative FACS plots and quantification showing the frequency of MEF that uptake CFSE-labeled ECVs in 24 hrs *in vitro* (*n* = 4 independent experiments). Data is represented as mean ± SEM. (**E**) Analysis of Cre RNA in MEF that were treated with B16-GFP-Cre ECVs for 24 hrs by qPCR. Data were normalized against *Hprt* (*n* = 3 independent experiments). Data is represented as mean ± SEM. (**F**) Quantification of tdTomato expression in reporter MEF that were cultured alone (control) or indirectly with B16-GFP-Cre cells (separated by a membrane with 0.4μm pores) for up to 4 days (*n* = 4 independent experiments). Data is represented as mean ± SEM. See also Supplementary Figure 1.

Previous reports have demonstrated that Cre activity can be transferred between cells via ECVs [33, 34, 35]. We therefore sought to determine if ECVs alone are responsible for mediating the rapid Cre transfer we observed in our model system. We purified ECVs from the conditioned media of B16-GFP-Cre cells via differential ultracentrifugation and verified their identity using electron microscopy. Our isolates consisted of vesicles that were around 100 nm in diameter with a “cup-shaped morphology” typically associated with small vesicles (Supplementary Figure 1). We next examined Cre protein and transcript in the ECVs. We found that while the amount of Cre protein in ten μg of B16-GFP-Cre ECVs was below the detection level of western blotting (Figure 2A), Cre transcript was highly enriched in B16-GFP-Cre ECVs relative to ECVs from control B16 cells (Figure 2B). This result matches previously published reports showing that Cre RNA, but not protein, can be detected in ECVs derived from Cre-expressing cancer cells [34].

### ECVs do not mediate the rapid transfer of Cre between B16-GFP-Cre cells and MEFs

Next, we sought to determine if B16-GFP-Cre ECVs contain Cre activity. We administered varying concentrations of ECVs onto reporter MEFs for up to three days and measured tdTomato expression by FACS. In any condition tested, we were unable to detect tdTomato^+^ cells, suggesting that Cre^+^ ECVs alone are not sufficient to activate the reporter locus (Figure 2C). This was not due to the inability of MEFs to uptake ECVs, since CFSE-labeled exosomes were taken up by MEFs in a dose-dependent manner (Figure 2D). Despite the transfer of B16-GFP-Cre ECVs into MEFs, we were unable to detect an increase in Cre transcript in ECV-treated MEFs to any significance (Figure 2E). These results suggest that even though ECVs can be taken up by MEF and contain Cre RNA, they do not transfer *enough* Cre RNA to generate bioactive Cre protein in the target cell, and as a result, they alone are not sufficient to activate the reporter locus.

Since it is possible that the bulk addition of ultracentrifugation-isolated ECVs does not recapitulate physiological ECV release, we sought to establish if physiologically secreted ECVs were sufficient to activate the reporter locus in MEF. We co-cultured reporter MEF and B16-GFP-Cre cells for up to four days with the cells separated by a transwell insert with 0.4 μm pores so that ECVs could pass through the membrane but cells could not. In these conditions, we were unable to detect an increase in tdTomato^+^ MEF compared to control conditions (Figure 2F). Based on these cumulative results, we conclude that in our model system, the rapid exchange of Cre between cancer cells and non-cancer cells is not mediated by ECVs but instead by some other mechanism.

### The rapid transfer of Cre between B16-GFP-Cre cells and MEF occurs via cell-cell fusion

**Figure 3:**
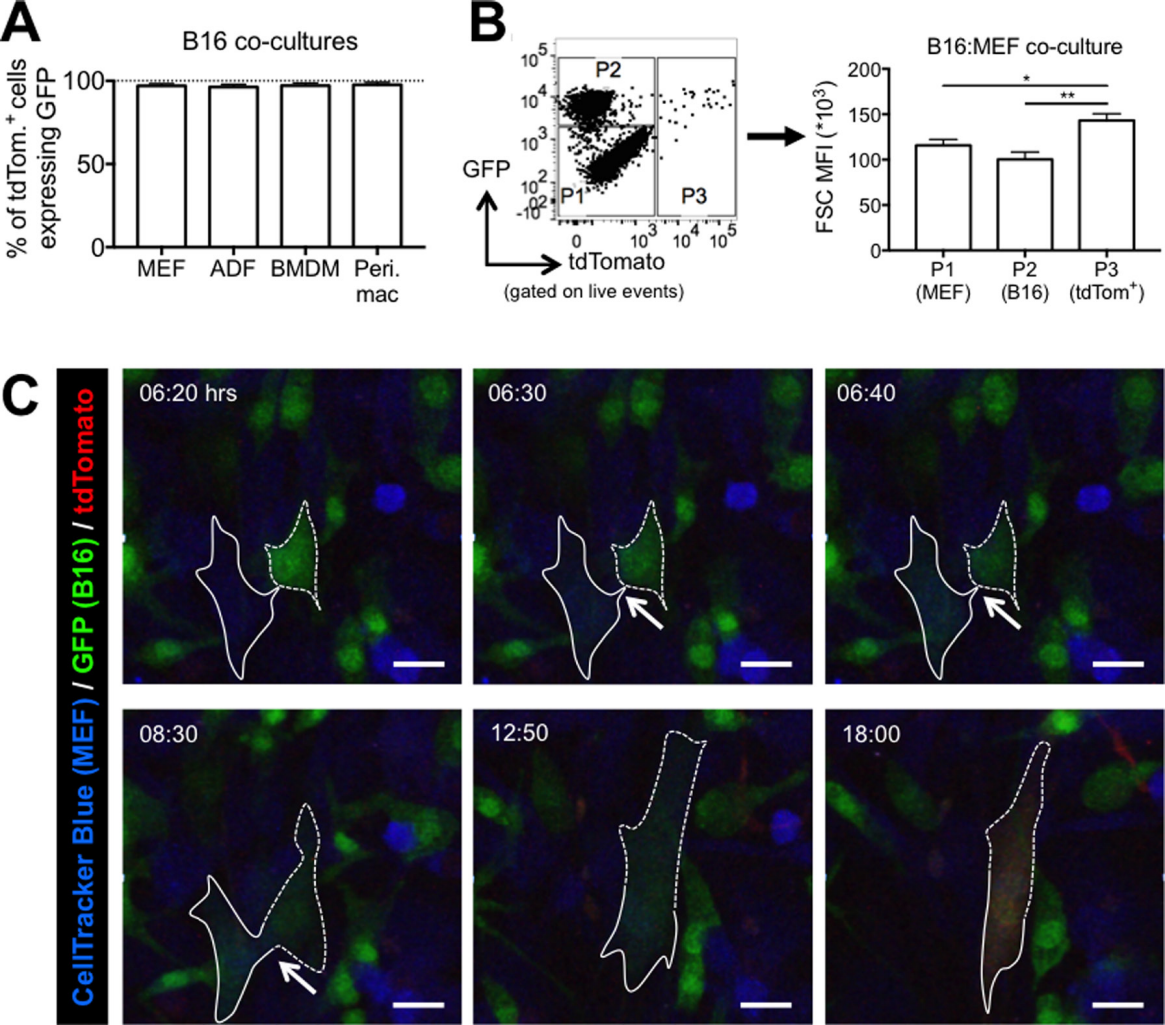
Cell-cell fusion mediates the rapid transfer of bioactive Cre from B16 melanoma cells to non-cancer cells *in vitro* (**A**) Quantification of GFP expression in tdTomato^+^ cells from 48 hour co-cultures of B16-GFP-Cre cells with various reporter cells (*n* = 3–4 independent experiments). Data is represented as mean ± SEM. (**B**) Quantification of FSC MFI of three populations of cells from a 24 hour B16:MEF co-culture: P1 = GFP^–^, tdTomato^-^ (MEF); P2 = GFP^+^ (B16 cells); P3 = tdTomato^+^ (MEF that received bioactive Cre) (*n* = 7 independent experiments). Data is represented as mean ± SEM. See also Supplementary Figure 2. (**C**) Stills from confocal imaging movie (Video 1) of B16:MEF co-culture showing a CellTracker Blue-labeled reporter MEF (outlined in solid white line) turn green and then red after fusing with a B16-GFP-Cre cell (outlined in dashed white line). Arrows indicate the area of contact between the MEF and B16 cell that ultimately fuse and start expressing tdTomato at 18:00 hrs. See also Supplementary Videos 1–5.

Two intriguing observations led us to hypothesize that Cre transfer may occur via cell-cell fusion. First, we noticed that in all B16 co-cultures tested, nearly all tdTomato^+^ cells also expressed GFP (Figure 3A). Second, we saw that tdTomato^+^ cells from B16:MEF co-cultures had a significantly higher forward scatter (FSC), which is a read-out for cell size, than B16 cells (1.43-fold, *p <* 0.01) and MEF (1.24-fold, *p <* 0.05), indicating that they are larger in size (Figure 3B). In fact, tdTomato^+^ cells from every B16 co-culture tested had a higher FSC than both the reporter cells and B16-GFP-Cre cells (Supplementary Figure 2). Based on these observations, we hypothesized that Cre transfer between cancer and non-cancer cells may occur via cell-cell fusion.

To investigate this hypothesis further, we performed live-cell confocal imaging on B16-GFP-Cre cells co-cultured with CellTracker Blue-labeled reporter MEF, allowing us to definitively identify the origin of tdTomato^+^ cells. B16 cells were added to adherent MEF, and video recording was initiated after 2 hours. As seen in Supplementary Video 1 and Figure 3C, we observed a MEF (outlined in a solid white line) interacting with a B16 cell (outlined in a dashed white line) at 6:20 hrs. Over the course of less than 20 minutes, the MEF suddenly attained GFP expression, while the intensity of the GFP signal from the B16 cell diminished, suggesting that at this moment, the cytosol of each cell physically connected, allowing the GFP from the B16 cell to diffuse into the cytosol of the MEF. Following this event, the cells remained physically connected for the next several hours (indicated by the white arrows), and by 12:50 hrs, they joined together and appeared to fuse into a single cell, noticeably larger than those around it. By 18:00 hrs, the heterokaryon began expressing tdTomato (Figure 3C) demonstrating that a tdTomato^+^ cell is *both* a MEF and a B16 cell.

We observed many other instances of cell-cell fusion mediating Cre transfer between MEF and B16 cells. In one example, the fusion event happened nearly immediately after initiating video recording (Supplementary Video 2). In another, the cells had already fused prior to starting the video, evident by the appearance of a GFP^+^/CellTracker Blue^+^ cell at the beginning of the video that eventually turned red (Supplementary Video 3). We also observed a large tdTomato^+^ cell undergoing what appeared to be programmed cell death after approximately 32 hrs, suggesting that these hybrid cells may be unstable (Supplementary Video 4). Finally, using a different experimental setup where MEF were left unlabeled, we observed a tdTomato^+^ cell originate from a GFP^+^ cell (Supplementary Video 5).

### B16xMEF hybrids contain B16-restricted DNA but do *not* maintain expression of B16-restricted GFP

**Figure 4:**
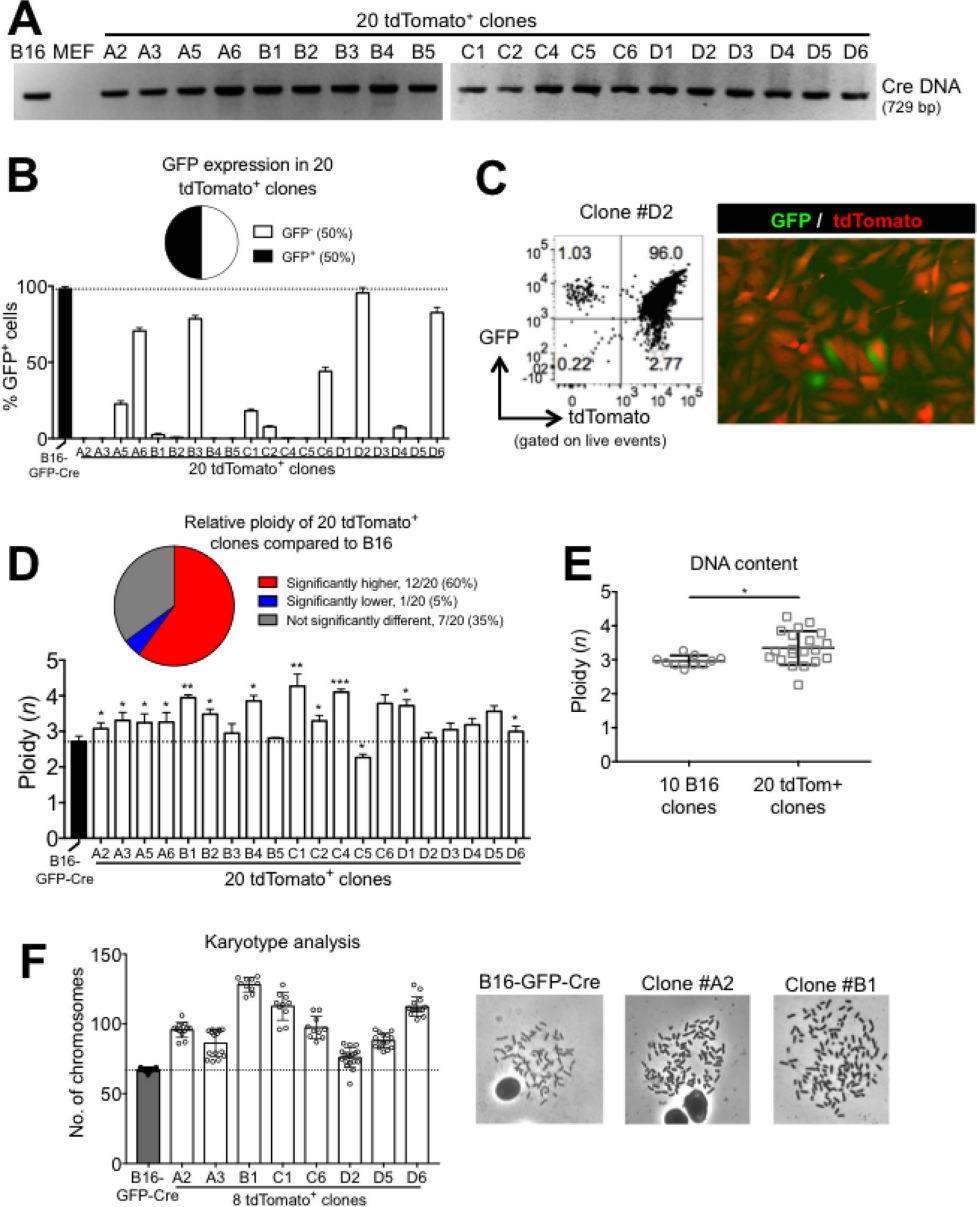
B16xMEF hybrids stably maintain B16-restricted DNA, do *not* maintain expression of B16-Restricted GFP, and are hyperploid (**A**) Analysis of Cre DNA in B16-GFP-Cre, MEF, and twenty tdTomato^+^ clones by PCR. (**B**) Quantification of GFP expression in B16-GFP-Cre and twenty tdTomato^+^ clonal cell lines measured by FACS (*n* = 3 independent experiments). Data is represented as mean ± SEM. (**C**) FACS plot and fluorescent micrograph showing GFP and tdTomato expression in one of the three tdTomato^+^ clones (#D2) that showed *loss* of tdTomato expression. (**D**) Quantification of DNA content of B16-GFP-Cre and twenty tdTomato^+^ cell lines by FACS. Ploidy was determined by normalizing the 7-AAD MFI of each cell line relative to MEF, which was set at 2*n* (*n* = 3 independent experiments). Data are represented as mean ± SEM. See also Supplementry Figure 3. (**E**) Comparison of the average DNA content in ten B16-GFP-Cre clones and twenty tdTomato^+^ clones as determined by FACS (*n* = 2–3 independent experiments per data point). Data is represented as mean ± SEM. (**F**) Quantification of chromosome number in B16-GFP-Cre cells and eight tdTomato^+^ clonal cell lines by karyotyping (*n* = 15–20 metaphase spreads per group). Shown is mean ± SD. Representative metaphase spreads of B16-GFP-Cre and two tdTomato^+^ clones are shown on the right.

Spontaneously fused cancer-cell:normal cell hybrids have not been studied extensively. Having established a simple and robust model of cell fusion, we further characterized the fused cells by generating clonal cell lines. Limiting dilution cloning of sorted tdTomato^+^ cells was performed from a 24 hour B16:MEF co-culture. After the 4th passage (about 6-8 weeks in culture), we extracted DNA from each of the clones and probed for the presence of Cre DNA by PCR. We found that 100% (20/20) of the clones contained Cre DNA, which is restricted to B16 cells and absent in MEF (Figure 4A). Since each clone also maintained high expression of MEF-restricted tdTomato (data not shown), this strongly suggests that the tdTomato^+^ clones originated from B16xMEF hybrid cells. Interestingly, despite the fact that 100% of the tdTomato^+^ clones contained B16-restricted DNA, only 50% (10/20) maintained expression of B16-restricted GFP (Figure 4B). In addition, 15% (3/20) of the clones lost expression of tdTomato in a small sub-population of cells (Figure 4C). These results illustrate how despite inheriting DNA from two different cells, the hybrid clones do not necessarily express the same genes as both parent cells. This suggests that genetic silencing and/or deletion likely occurred during the clonal expansion of the hybrid clones. Indeed, epigenetic reprogramming is a common hallmark of hybrid cells [36]. The silencing and/or loss of genes [37] and even whole chromosomes [38] have been documented to occur following a cell fusion event, so this result matches previous findings.

### B16xMEF hybrids are hyperploid

Next, we quantified the DNA content of the tdTomato^+^ clones and B16-GFP-Cre cells using two independent techniques. By FACS, we observed that 60% (12/20) of the tdTomato^+^ clones contained significantly more DNA than B16-GFP-Cre cells (which are already known to be hyperploid [39]), only 5% (1/20) contained significantly less, and 35% (7/20) were not significantly different (Figure 4D and Supplementary Figure 3). We independently generated ten B16-GFP-Cre clonal cell lines and found that the average DNA content of the twenty tdTomato^+^ clones was significantly higher (1.13-fold) than the ten B16-GFP-Cre clones (Figure 4E). Similarly, by karyotype analysis we observed that 100% (8/8) of the tdTomato^+^ clones tested contained more chromosomes than B16-GFP-Cre cells. In fact, one clone (#B1) averaged 128.1 chromosomes per cell, which is 3.20-fold higher than a non-transformed cell and 1.88-fold higher than B16 (Figure 4F). These results clearly show that tdTomato^+^ clones contain more DNA than B16 and provide strong evidence that DNA is pooled between MEF and B16 cells during a cell fusion event.

### B16xMEF hybrids express both B16- and MEF-restricted genes

**Figure 5:**
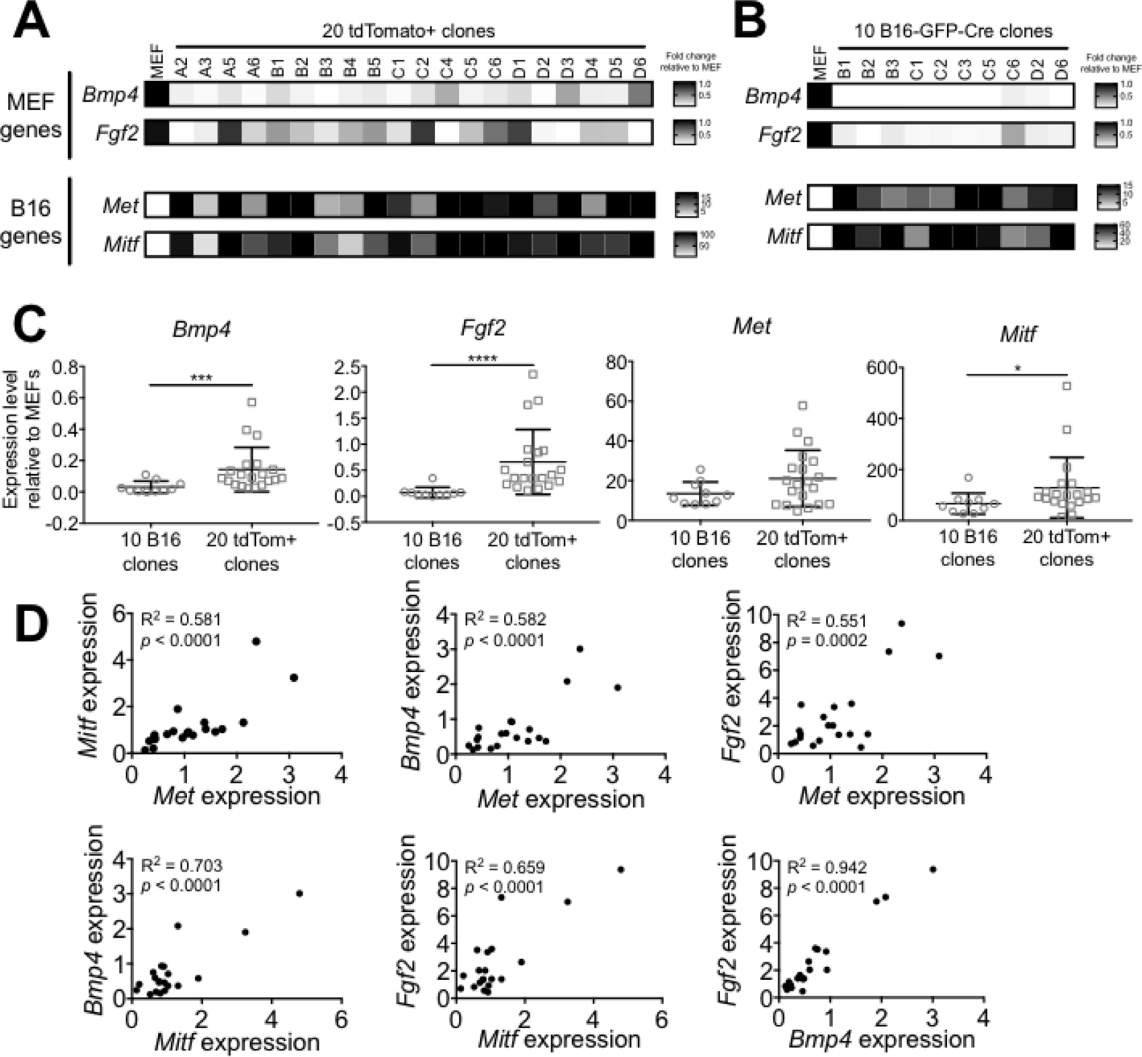
B16xMEF hybrids express B16- and MEF-restricted genes (**A**) Heat map showing the relative expression level of two candidate “MEF genes” (*Bmp4, Fgf2*) and two candidate “B16 genes” (*Met*, *Mitf*) in MEF and twenty tdTomato^+^ clones. The expression level of each gene is shown as relative to MEF and normalized against *Hprt* (*n* = 2 independent experiments). (**B**) Heat map showing the expression level of *Bmp4, Fgf2, Met*, and *Mitf* in ten B16-GFP-Cre clones relative to MEF and normalized against *Hprt* (*n* = 2 independent experiments). (**C**) Comparison of the average expression level of *Bmp4, Fgf2, Met*, and *Mitf* in ten B16-GFP-Cre clones and twenty tdTomato^+^ clones (*n* = 2–3 independent experiments per data point). Data is represented as mean ± SEM. See also Supplementary Figure 4. (**D**) Dots plots comparing the expression level of *Bmp4, Fgf2, Met*, and *Mitf* (relative to B16-GFP-Cre) in twenty tdTomato^+^ clones.

To determine whether the combination of DNA resulting from cell fusion events could lead to functional changes in gene expression, we examined expression of candidate “MEF genes” (*Bmp4, Fgf2*) and “B16 genes” (*Met, Mitf*) in the tdTomato^+^ clones versus the B16-GFP-Cre clones and MEF by qPCR. Among the tdTomato^+^ clones, the variability in expression of both MEF- and B16 genes was very high: some clones expressed as much as or more of a given gene than B16 cells and MEF, while in other clones the genes were barely expressed (Figure 5A). By contrast, the ten B16-GFP-Cre clones homogenously expressed B16 genes and lacked expression of MEF genes (Figure 5B). On average, the expression of the MEF genes was significantly higher in the tdTomato^+^ clones than the B16 clones (*Bmp4*: 4.49-fold, *p* > 0.001 and *Fgf2*: 9.02-fold, *p* > 0.0001), and the expression of the B16 genes was at least as high in the tdTomato^+^ clones than the B16 clones (Figure 5C). This suggests that the hyperploidy resulting from DNA mixing allowed for gene expression patterns of both parent cells (in this case, MEF and B16) to be maintained in some daughter cells over many generations. We observed similar results using an expanded panel of genes specific for MEF (*Cd24a, Gas1*, *Sca1*) and B16 (*Trpm1*, *Tyr*) (Supplementary Figure 4).

We found that the variability in expression level of *Bmp4*, *Fgf2*, *Met*, and *Mitf* was significantly higher among the tdTomato^+^ clones compared to the B16-GFP-Cre clones at the α = 0.01 confidence level. This result implies that cell-cell fusion diversifies the gene expression profile of hybrid cells, increasing clonal heterogeneity at the populational level. Interestingly, we observed a strong correlation between the expression levels of each of the four genes among the twenty tdTomato^+^ clones (Figure 5D). This shows that the expression of MEF- or B16 genes is not mutually exclusive in hybrid cells, since certain tdTomato^+^ clones (like #C4, D3, and D6) express high levels of both B16- and MEF genes. This result provides evidence that cell-cell fusion not only acts as a mechanism of DNA exchange but also a modulator of gene expression in cancer.

### Cell-cell fusion promotes resistance against chemotherapeutics in B16 melanoma cells *in vitro*

**Figure 6:**
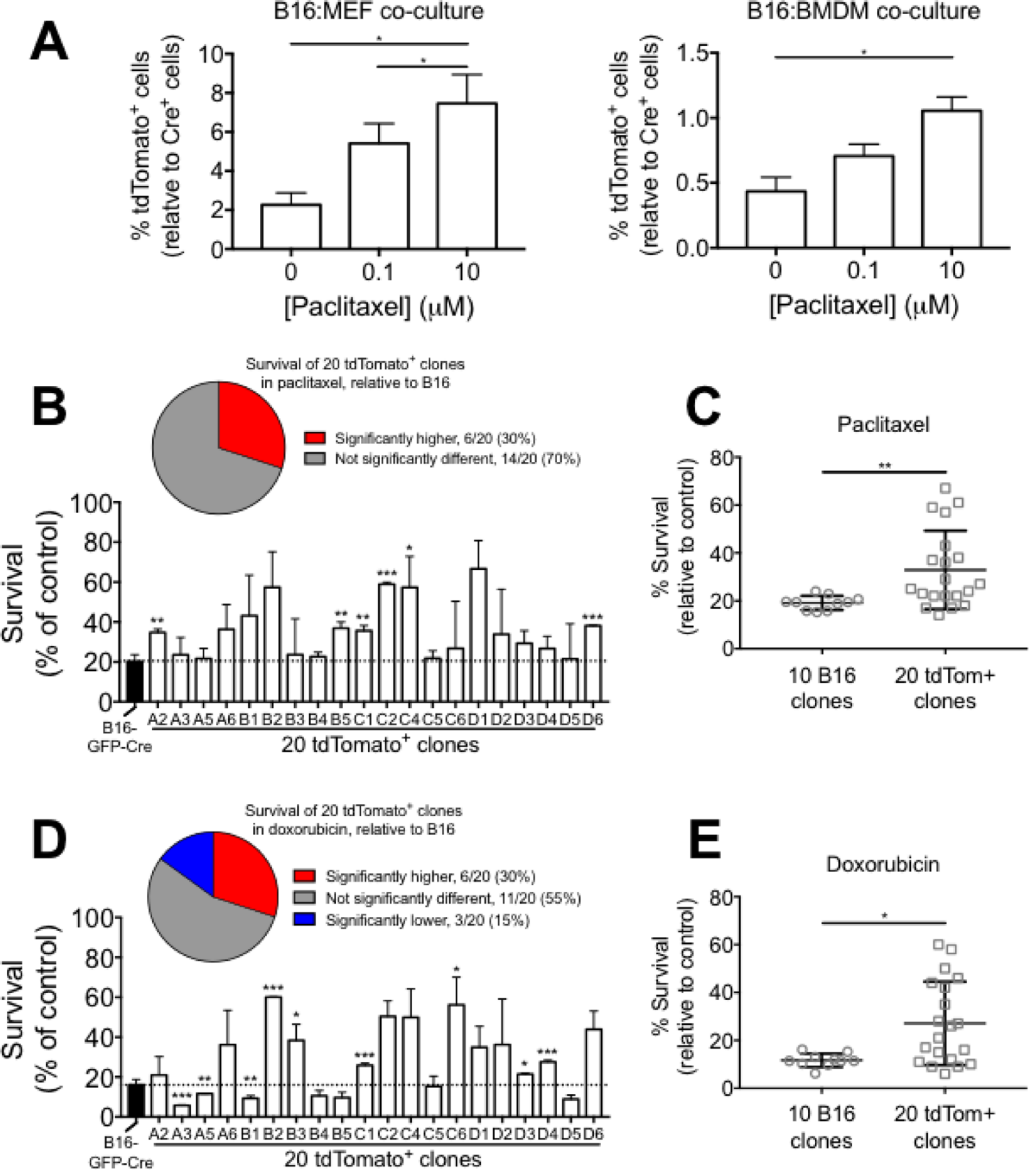
Cell-cell fusion promotes resistance to chemotherapeutics in B16 melanoma cells *in vitro* (**A**) Quantification of tdTomato^+^ cells in B16:MEF and B16:BMDM co-cultures grown together for 24 hrs and then treated with 0, 0.1, or 10 μM paclitaxel for 24 hrs (*n* = 3 independent experiments). Data is represented as mean ± SEM. (**B**) Bar graph showing survival of B16-GFP-Cre and twenty tdTomato^+^ clones grown in the presence of paclitaxel (10 μM) for 24 hrs relative to control (*n* = 2–3 independent experiments). Data are represented as mean ± SEM. (**C**) Comparison of the average survival rate of ten B16-GFP-Cre clones and twenty tdTomato^+^ clones in the presence of 10 μM paclitaxel for 24 hrs (*n* = 2–3 independent experiments). Data are represented as mean ± SEM. (**D**) Bar graph showing survival of B16-GFP-Cre and twenty tdTomato^+^ clones grown in the presence of doxorubicin (10 μM) for 24 hrs relative to control (*n* = 2–3 independent experiments). Data are represented as mean ± SEM. (**E**) Comparison of the average survival rate of ten B16-GFP-Cre clones and twenty tdTomato^+^ clones in the presence of 10 μM doxorubicin for 24 hrs (*n* = 2–3 independent experiments). Data are represented as mean ± SEM.

Previous reports have demonstrated that cell-cell fusion induces chemoresistance in cancer [12, 18, 40, 41]. However, many studies have used artificial fusion methods (electroporation or fusion-inducing chemicals) to generate hybrid cells and therefore may not reflect true physiology. We sought to employ our more physiological model to ask if cell-cell fusion promotes chemoresistance in melanoma. We grew co-cultures of B16-GFP-Cre cells with reporter target cells (either MEF or BMDM) for 24 hours, treated the co-cultures with various concentrations of paclitaxel for 24 hours, and then measured the frequency of tdTomato^+^ cells by FACS. We found that the frequency of tdTomato^+^ cells was significantly enriched in both B16:MEF and B16:BMDM co-cultures that were exposed to paclitaxel relative to control conditions (Figure 6A) suggesting that B16xMEF and B16xBMDM hybrids are more resistant to paclitaxel than B16 cells. Alternatively, it is possible that chemotherapy induces cell fusion, as has previously been reported [42].

We also tested the sensitivity of each of the twenty tdTomato^+^ clones to paclitaxel and compared against B16-GFP-Cre. In the presence of 10 μM paclitaxel for 24 hours, we found that 30% (6/20) of the tdTomato^+^ clones had a significantly higher survival rate than B16-GFP-Cre, and 70% (14/20) were not significantly different (Figure 6B). On average, the twenty tdTomato^+^ clones were significantly more resistant (1.72-fold, *p* < 0.01) to paclitaxel than the ten B16-GFP-Cre clones (Figure 6C). We also found that the variability in resistance to paclitaxel was significantly higher in the tdTomato^+^ clones than the B16-GFP-Cre clones at a confidence level of α = 0.01. We performed the same experiment using a second chemotherapeutic, doxorubicin. In the presence of 10 μM doxorubicin for 24 hours, we found that 30% (6/20) of the tdTomato^+^ clones had a significantly higher survival rate than B16-GFP-Cre, 15% (3/20) had a significantly lower survival rate, and 55% (11/20) were not significantly different (Figure 6D). On average, the twenty tdTomato^+^ clones were significantly more resistant (2.32-fold, *p* < 0.05) to doxorubicin than the ten B16-GFP-Cre clones (Figure 6E). Similar to paclitaxel, we found that the variability in doxorubicin resistance was significantly higher in the tdTomato^+^ clones than the B16-GFP-Cre clones at a confidence level of α = 0.01. Together, these results demonstrate that cell-cell fusion promotes chemoresistance at both the single cell- and population level, and show that clones derived from hybrid cells can vary greatly in terms of survival against paclitaxel and doxorubicin.

### Cre transfer from B16 melanoma cells to non-cancer cells occurs *in vivo* within the tumor microenvironment

**Figure 7:**
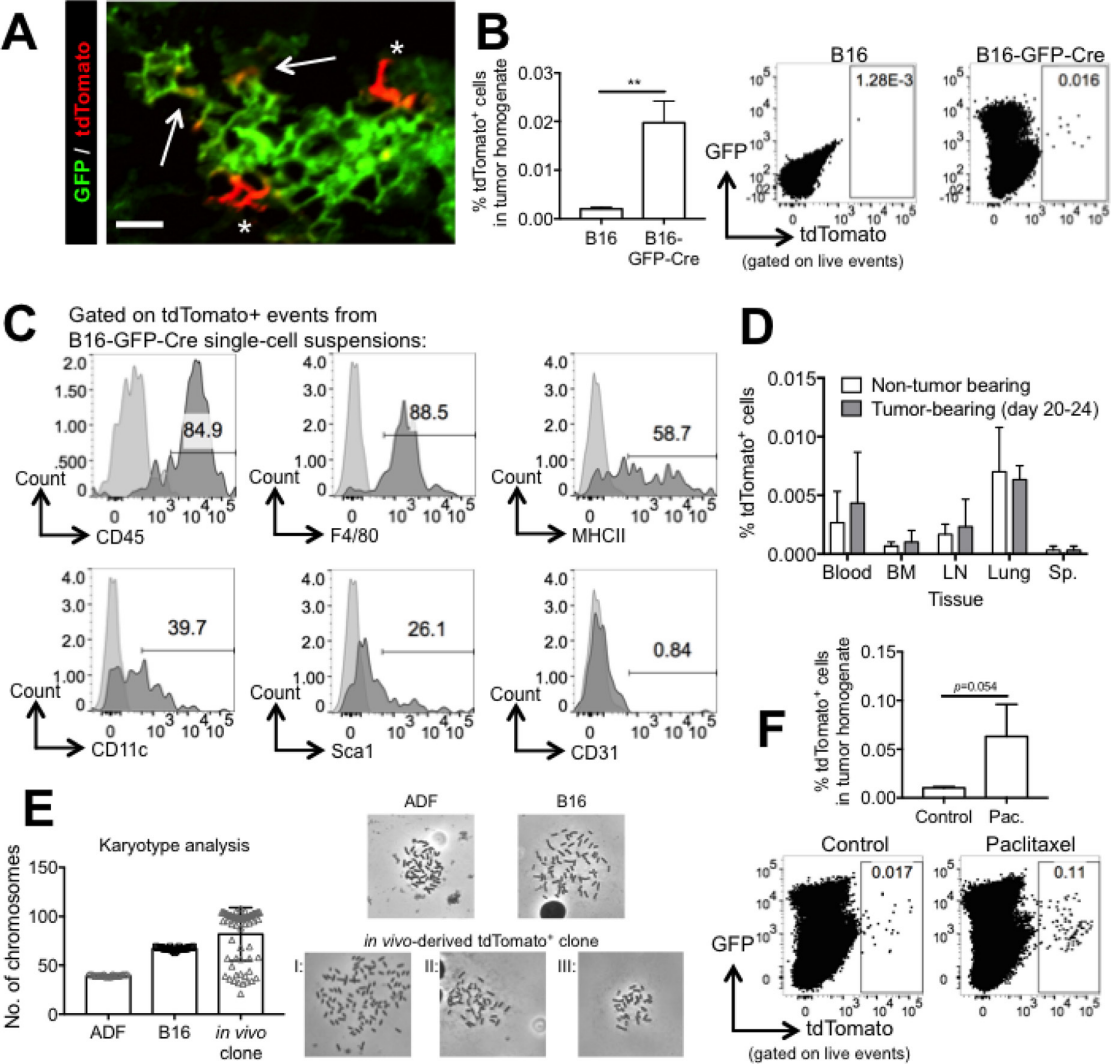
Cre transfer from B16 melanoma cells to non-cancer cells occurs *in vivo* and promotes survival in the presence of paclitaxel (**A**) Representative confocal micrograph showing GFP and tdTomato expression in a flash-frozen B16-GFP-Cre tumor grown in a reporter mouse for 21 days. Asterisks indicate cells expressing tdTomato, and arrows indicate cells expressing both tdTomato and GFP. Scale bar equals 50 μM. (**B**) Quantification and representative FACS plots of tdTomato expression in single-cell suspensions of B16 or B16-GFP-Cre tumors that were grown in reporter mice for 18-21 days. *n* = 4 (B16) or 10 (B16-GFP-Cre) mice. Data are represented as mean ± SEM. (**C**) FACS analysis of CD45, F4/80, MHCII, CD11c, Sca-1, and CD31 expression on tdTomato^+^ cells from B16-GFP-Cre single-cell suspensions. Histograms in black were obtained using fluorescently-labeled antibodies against each of the antigens listed, and histograms in light grey were obtained using isotype control antibodies. (**D**) Quantification of tdTomato expression in single-cell suspensions of various tissues including blood, bone marrow (BM), lymph nodes (LN), lung, and spleen (Sp.) from reporter mice bearing B16-GFP-Cre tumors for 0 or 20–24 days (*n* = 4 mice per group). Data are represented as mean ± SEM. (**E**) Karyotype analysis of adult dermal fibroblasts (ADF), B16-GFP-Cre, and an *in vivo*-derived tdTomato^+^ clonal cell line. *n* = 10 (ADF and B16) or 66 (*in vivo* clone) metaphase spreads per group. Shown is mean ± SD. Representative metaphase spreads of ADF, B16-GFP-Cre and the three types of karyotypes observed in the *in vivo* clone are shown on the right. See also Supplementary Figure 5. (**F**) Representative FACS plots and quantification of tdTomato expression in single-cell suspensions of B16-GFP-Cre tumors grown in reporter mice that were treated with HBSS or paclitaxel. Tumors were harvested after reaching a size of 10 × 10 mm^2^ (*n* = 6 mice per group). Data are represented as mean ± SEM. See also Supplementary Figure 6.

Having demonstrated that Cre transfer occurs *in vitro*, we sought to establish whether this phenomenon also occurs *in vivo*. We injected B16-GFP-Cre melanoma cells (1e6 cells, s.c.) into reporter mice, and after tumors reached 10 × 10 mm in size (about 18–21 days), we harvested the tumors for analysis of tdTomato^+^ cells by microscopy and FACS. In sections of flash-frozen B16-GFP-Cre tumors, we observed tdTomato^+^ cells (asterisks), demonstrating that Cre transfer does occur *in vivo* (Figure 7A). We noticed that some of the tdTomato^+^ cells also expressed GFP (arrows), an observation that supports the hypothesis that *in vivo* Cre transfer may occur via cell-cell fusion. By FACS, we found that the frequency of tdTomato^+^ cells was significantly higher in single-cell suspensions of B16-GFP-Cre tumors compared to control B16 tumors that did not express Cre, indicating that FACS is sensitive enough to detect and quantify Cre transfer *in vivo* (Figure 7B). However, it must be noted that the frequency of tdTomato^+^ cells in B16-GFP-Cre tumors was very low (<0.02% of all cells), indicating that *in vivo* Cre transfer in B16 melanoma is a rare phenomenon or that tdTomato^+^ cells do not remain viable after receiving B16-derived Cre.

We next sought to identify which cells had received B16-derived Cre by characterizing the surface markers expressed by tdTomato^+^ cells in single-cell suspensions of B16-GFP-Cre tumors. By FACS, we found that a majority (but not all) of tdTomato^+^ cells expressed CD45, indicating that both hematopoietic and, to a lesser degree, non-hematopoietic cells can uptake Cre. The majority of tdTomato^+^ cells expressed F4/80, suggesting that the immune cells that uptake Cre are macrophages. This result matches previous results showing macrophages are capable of fusing with cancer cells [11, 12, 14, 43]. A small portion of tdTomato^+^ cells expressed additional immune-related markers (MHCII, CD11c). We noticed a small population of tdTomato^+^ cells expressing the stem cell marker Sca-1, suggesting that progenitor cells within the tumor microenvironment can uptake B16-derived Cre. We did not detect expression of the endothelial marker CD31 on any tdTomato^+^ cells (Figure 7C).

To determine if Cre can be transferred to sites distal from the tumor, we used FACS to quantify the frequency of tdTomato^+^ cells in a variety of different tissues (blood, bone marrow, lymph nodes, lung, spleen) from control, non-tumor bearing reporter mice or reporter mice bearing B16-GFP-Cre tumors for 18–21 days. In all tissues tested, we were unable to detect significantly more tdTomato^+^ cells in tumor-bearing compared to control mice (Figure 7D). This result demonstrates that in our model system, the amount of Cre transfer to distal sites is below the detection limit of FACS, suggesting it is an even rarer phenomena than local Cre transfer or does not occur at all.

### Cell-cell fusion between B16 melanoma cells and non-cancer cells occurs *in vivo*

Since we demonstrated that cell-cell fusion can mediate Cre transfer *in vitro*, we sought to determine if this phenomenon occurs *in vivo*. We generated an “*in vivo*-derived” tdTomato^+^ clonal cell line using limited dilution cloning on tdTomato^+^ cells that were sorted from a twenty day B16-GFP-Cre tumor single-cell suspension. After four passages (6–8 weeks in culture), we performed karyotype analysis to quantify the number of chromosomes in the *in vivo*-derived clone, B16-GFP-Cre cells, and a non-transformed cell, adult dermal fibroblasts (ADF). Per cell, ADF had 39.7 ± 0.7 chromosomes, B16-GFP-Cre cells had 67.1 ± 1.3, and the *in vivo*-derived clone had 81.7 ± 27.1. Cells from the *in vivo*-derived clone contained between 21 to 105 chromosomes, and we observed 3 different groups within this clone based on chromosome number. Most cells (group I, 68%) had greater than 90 chromosomes per cell, 17% of cells had 40-90 chromosomes (group II), and 19% of cells had less than 40 chromosomes (group III) (Figure 7E). These results not only demonstrate that the *in vivo*-derived clone has more DNA than B16 and non-transformed cells, but also highlight the immense variability in the genome size of this clone. This is likely a reflection of genomic instability, which is a hallmark of hybrid cells with high amounts of DNA [38, 44, 45]. Together, these observations show that the *in vivo*-derived clone is likely a hybrid cell and support the hypothesis that cell-cell fusion can occur *in vivo* and induce clonal heterogeneity. In addition, we observed that a sub-population of the *in vivo*–derived tdTomato^+^ clone expressed GFP, demonstrating that the tdTomato^+^ clone originated from a cell that also contained B16-restricted GFP (Supplementary Figure 5A). We also saw that the *in vivo*-derived clone had a higher FSC MFI than B16-GFP-Cre cells, demonstrating that these cells are larger in size (Supplementary Figure 5B).

### Cre transfer between B16 melanoma cells and non-cancer cells promotes survival in the presence of paclitaxel *in vivo*

To demonstrate that the presumed fused cells displayed higher chemoresistance *in vivo*, we injected reporter mice with B16-GFP-Cre cells (1e6 cells s.c.) and treated them with paclitaxel or HBSS (control) for 15 days. By day 17, paclitaxel treatment had reduced the size of tumors by an average of 60% (Supplementary Figure 6). After reaching a size of 10 × 10 mm, we harvested tumors and analyzed tumor single-cell suspensions for the frequency of tdTomato^+^ cells. We found that tdTomato^+^ cells were enriched 7.4-fold in paclitaxel-treated compared to control tumors (*p* = 0.054, Figure 7F). This result demonstrates that tdTomato^+^ cells are more resistant than B16 cells *in vivo* and supports the general hypothesis that chemoresistance in cancer is enhanced by cell-cell fusion.

## DISCUSSION

Cancer cell heterogeneity forms the substrate for chemoresistance, metastasis, and cancer progression [46, 47]. Cancers possess abundant clonotypes of cells that can be selected by external forces including chemotherapy, radiotherapy, and immune therapy. Although the driving force of cancer heterogeneity has been suggested to derive from accumulated spontaneous and random mutagenic events [48], it is also clear that during the process of transformation, whole-scale genomic alterations have occurred [49]. Indeed, nearly all cancers display aneuploidy, a result that has been attributed to a “crisis event” that occurs early during carcinogenesis [50, 51]. It is not clear what the “crisis” entails, and indeed, the mechanism by which cancers become aneuploid has still not been fully defined [52]. Here, we have found that cancer cells spontaneously fuse with normal cells to form hybrid hyperploid cells that we postulate contributes to some of the heterogeneity observed in cancer [45, 53, 54].

We observed cell-cell fusion using a Cre-*loxP* system that we had originally developed to track the exchange of molecular information between cancer and non-cancer cells. In this model system, we found that cancer cells expressing Cre could induce *loxP* recombination in normal reporter cells after direct co-culture, rendering the reporter cells red via expression of a tdTomato reporter gene. Surprisingly, the red cells all displayed hyperploidy and were found to result from a cell fusion event. The most efficient Cre transfer occurred between melanoma cells and fibroblasts, where 2–6% of cells expressed tdTomato after 48 hours. We observed *in vivo* Cre transfer from melanoma cells to non-cancer cells within the tumor microenvironment, but only at a very low frequency (∼0.02% of cells). These numbers are in line with reports that suggest only about 1% of tumor cells fuse *in vivo* [17] and the hypothesis that only about 1% of fused cells actually survive/ proliferate [55], so in total only about 0.01% of tumor cells are hybrids. We did not observe Cre transfer to sites distal from the tumor, including spleen, lymph nodes, lung, bone marrow, or blood. This result, although negative, infers that ECVs were not mediating Cre transfer in our model system.

Two recent publications using a similar Cre-*loxP* system concluded that Cre was transmitted to reporter cells via ECVs [34, 35]. We took care to demonstrate that this was not occurring in our experimental system. We showed that purified ECVs could not transfer enough Cre to target cells to induce *loxP* recombination. This finding cannot be attributed to a lack of uptake of ECVs by target cells, since we observed uptake of CFSE-labeled ECVs by MEF, nor by an absence of Cre in ECVs, since we detected Cre transcript by PCR. Rather, it appears that not enough Cre is transferred from ECVs to target cells in order to induce reporter activation. This could be because our Cre expression system lacks the “ECV targeting motif” used by [34], or because we expressed Cre bi-cistronically with GFP, making it a larger transcript than if Cre were expressed alone. However, since we do see Cre transcript significantly enriched in ECVs from Cre expressing cells, we do not think these are likely explanations. Moreover, when Zomer *et al.* attempted this experiment, they also could not demonstrate functional Cre transfer via direct administration of ECVs to any significance [35]. Therefore, we conclude that the rapid Cre transfer we observed is not mediated by ECVs.

We report here that Cre exchange is mediated by cell-cell fusion, an observation that is supported by the fact that 100% of the tdTomato^+^ clonal cell lines were hyperploid and contained DNA from both cancer and non-cancer cells. Our gene expression data also back up this hypothesis by showing that our hybrid clones express genes from both parent cells (B16 and MEF), mirroring previous studies and showing that there are functional consequences of DNA exchange that occurs during cell-cell fusion [20, 56]. Furthermore, these findings support the broad hypothesis that cell-cell fusion could mediate aneuploidy in cancer, since all of the hybrid-derived clonal cell lines created in this study contained atypical numbers of chromosomes [45, 57]. This result highlights the efficiency of cell-cell fusion as a mechanism by which cancer cells can attain aneuploid numbers of chromosomes in the absence of cytokinesis failure. However, it must be noted that the process of cell-cell fusion is distinct from entosis, which has also been shown to induce aneuploidy in human cancers [58].

Importantly, we also show here that cell-cell fusion increases the phenotypic heterogeneity of cancer cells. In this sense, fusion acts as a driver of clonal diversification in cancer. This result matches previous reports highlighting the unique involvement of cell-cell fusion in enhancing phenotypic diversification of cancer [8, 18]. Evidence for this comes from comparing variance among hybrid vs. non-hybrid B16 clones in terms of DNA content, gene expression, and resistance to chemotherapeutics. For each parameter tested, we observed significantly more variance in the hybrid clones compared to non-hybrid clones. This result clearly illustrates that the process of cell-cell fusion imparts a higher degree of phenotypic variability to daughter cells. These observations support the hypothesis that cell-cell fusion is useful to an evolving cancer cell population by more easily creating a cell with novel properties that can survive a given selective pressure. In fact, we speculate that cell fusion is perhaps the most efficient way to create heterogeneity in a cancer cell population.

Previous reports have shown that cell-cell fusion can modulate various properties of cancer cells including resistance to chemotherapy. A recent study using metastatic colon carcinoma showed that cell-cell fusion occurs *in vivo* and is involved in the appearance of tumor cells that were resistant to both 5-fluorouracil (5-FU) and oxaliplapin [41]. Another study using breast cancer cells showed that cell lines derived from breast cancer/epithelial cell fusion exhibited increased resistance to several chemotherapeutics (5-FU, doxorubicin, etoposidase, and paclitaxel) compared to parental breast cancer cell lines [40]. Similarly, we found that our hybrid B16 cell lines were more resistant to two different chemotherapeutics compared to non-fused B16 cells *in vitro*. Further, we found a higher relative frequency of tdTomato^+^ cells in B16 tumors that were treated with chemotherapy compared to untreated control tumors. This finding mirrors a recent report showing that chemotherapy increased tumor cell hybridization *in vivo* [42]. Together, these data support the hypothesis that cell-cell fusion contributes to tumor progression at least in part by allowing for a higher degree of heterogeneity, which in the presence of a selective pressure like chemotherapy, results in a higher likelihood that at least one cell will have mutated around it. In this regard, our data adds to the growing list of evidence that cell-cell fusion could have functional relevance in cancer progression.

## MATERIALS AND METHODS

### Contact for reagent and resource sharing

Requests for information and regents may be directed to and will be fulfilled by the Lead Contact, Dr. Jack Bui at the University of California, San Diego (jbui@ucsd.edu).

### Experimental model and subject details

#### Mice

Reporter mice (B6.Cg-*Gt(ROSA)26Sor*^*tm9(CAG-tdTomato)Hze*^/J) were purchased from Jackson Laboratories (Bar Harbor, ME, USA). 8–12 week old mice were age-and sex-matched for *in vivo* and *in vitro* experiments. All experiments involving mice were conducted under the animal protocol approved by the University of California, San Diego Institutional Animal Care and Use Committee (IACUC protocol #s06201).

### Cell lines and culture conditions

B16 melanoma cells and MCA sarcoma cells were cultured in RPMI 1640 supplemented with 10% (v/v) fetal bovine serum, 1 mM sodium pyruvate, 0.0375% sodium bicarbonate, 5% (v/v) MEM Non-essential amino acids, 2 mM L-glutamine, 10 μg/ml ciprofloxacin, and 56 μM 2-mercaptoethanol. B16.F10 cells were kindly provided by Dr. David Lyden and MCA sarcoma cell lines (4862, 6727, 9609, and 9614) were generated previously in our laboratory [31]. MDA-MB-231 breast cancer cell were grown in DMEM supplemented with 10% (v/v) fetal bovine serum and 1% (v/v) penicillin/streptomycin, and were kindly provided by Dr. Steve Gonias. Py117 breast cancer cells were grown in Ham’s F12K medium supplemented with 5% fetal calf serum, 2.5 ug/mL fungizone, 50 ug/mL gentamycin, and MITO+ and were generated previously in our laboratory [32]. All cells were grown and maintained in standard humidified tissue culture conditions (37°C with 5% CO_2_).

Each cancer cell line was engineered to express GFP-Cre by infection with a lentivirus encoding a bi-cistronic GFP-Cre expression cassette (GenTarget Inc., San Diego, CA, USA). After 48 hrs, GFP^+^ cells were sorted with a BD Aria II Cell Sorter (BD, Franklin Lakes, NJ, USA) and cloned using the limited dilution method. For each cell line, the clone with the highest expression of GFP was used. Reporter cells were all derived from transgenic mice harboring the “ROSA-LSL-tdTomato” reporter locus. BMDM were derived from BM cells cultured in the presence of 20% L929 conditioned media for 6 days. ADF and keratinocytes were generated as previously reported [59]. MEF were generated as described previously [60]. To generate tdTomato^+^ clonal cell lines, tdTomato^+^ cells were FACS sorted from a 48 hr B16:MEF co-culture using a BD Aria II Cell Sorter (BD, Franklin Lakes, NJ, USA) and cloned using the limited dilution method.

For B16 co-culture experiments, cells were seeded at a ratio of 1:10 (Cre-expressing cell:reporter cell). For co-culture experiments involving MCA sarcomas and breast cancer cells, cells were seeded at a ratio of 1:1 due to the lower expression of GFP-Cre in these cell lines. Co-culture experiments involving splenocytes were performed in the presence of human IL-2 (50 units/ml, Biolegend, San Diego, CA, USA). For transwell experiments, reporter MEF were seeded in the well of a 24-well plate and B16-GFP-Cre cells were seeded in the transwell insert with 0.4 μM pores (Corning Inc., Corning, NY, USA). The B16:MEF ratio was the same for the transwell experiment as it was for the direct co-culture (1:10).

### Method details

#### Tumor transplantation

Tumor cell lines were grown *in vitro*, harvested by trypsinization, washed three times, and resuspended at a final concentration of 5 × 10^6^ cells/mL in HBSS with Ca^2+^ and Mg^2+^. Two hundred microliters of cells (1 × 10^6^ total cells) were injected subcutaneously into the left flank of reporter mice. After tumors reached 10 × 10 mm in size (approximately 3 weeks), mice were sacrificed and tumors were processed into a single cell suspension by mechanical dicing/ collagenase digestion as previously described [31] and analyzed by FACS.

### Flow cytometry and antibodies

Reporter activation was measured by analysis of tdTomato expression in reporter cells by FACS. For analysis of *in vitro* co-cultures, cells were trypsinized, washed, and resuspended in FACS staining buffer (1X PBS with 1% FCS and 0.05% NaN_3_). For analysis of tumors grown *in vivo*, tumors were harvested after reaching 10 × 10 mm and processed into a single-cell suspension as described previously [31]. 7-aminoactinomycin D (7-AAD, Calbiochem, San Diego, CA, USA) was added immediately before FACS analysis at a final concentration of 1 μg/ml to stain and exclude dead cells from analysis.

For cell surface staining of *in vivo*-derived reporter^+^ cells, 1–2 × 10^6^ total cells were incubated for 20 minutes at 4°C with the following antibodies: APC-Cy7-conjugated anti-CD45, clone 30-F11 (Biolegend, San Diego, CA, USA), PE-Cy7-conjugated anti-F4/80, clone BM8 (Biolegend, San Diego, CA, USA), APC-conjugated anti-MHCII, clone M5/114.15.2 (Biolegend, San Diego, CA, USA), PE-Cy7-conjugated anti-CD11c, clone N418 (eBioscience, San Diego, CA, USA), PE-Cy7-conjugated anti-Sca1, clone D7 (Biolegend, San Diego, CA, USA), and AlexaFluor647-conjugated anti-CD31, clone 390 (Biolegend, San Diego, CA, USA). Surface staining was performed in the presence of 1 μg/ml F_c_ blocking anti-CD16/32 antibody. 7-AAD was added immediately before FACS analysis at a final concentration of 1 μg/ml. FACS was performed using a BD FACSCanto (BD, Franklin Lakes, NJ, USA) and data were analyzed using FlowJo software (Treestar, Ashland, OR, USA).

### ECV isolation

ECVs were isolated by differential ultracentrifugation as previously described [61]. Briefly, cells were grown for 48–72 hrs in media containing serum that had been depleted of ECVs by centrifugation for 70 minutes at 100,000 × g. The conditioned media was harvested and subjected to serial differential centrifugation steps to clear large and small debris as follows: 10 minutes at 500 × g (to remove large debris/ dead cells) followed by 20 minutes at 20,000 × g (to remove small debris/ apoptotic bodies). Next, the cleared conditioned media was spun for 70 minutes at 100,000 × g to pellet ECVs. The ECV pellet was resuspended in a large volume of HBSS and spun again for 70 minutes at 100,000 × g to wash soluble proteins from the ECVs. Finally, the washed pellet was resuspended in HBSS in a volume approximately 1/500th of the starting volume of conditioned media. The concentration of ECVs was determined by BCA assay (Thermo Fisher, Waltham, MA, USA). All ultracentrifugation steps were performed using a Beckman Avanti J-30I ultracentrifuge with a JA-30.50 Ti fixed-angle rotor (Beckman Coulter, Carlsbad, CA, USA).

### ECV characterization

The size and morphology of ECVs was evaluated by transmission electron microscopy using a previously described method [61]. ECVs were stained with 2% uranyl acetate for 1 minute, and grids were viewed using a JEOL 1200EX II (JOEL, Peabody, MA, USA) transmission electron microscope and photographed using a Gatan digital camera (Gatan, Pleasanton, CA, USA).

### Fluorescent labeling of ECVs

As described [62], ECVs were incubated with CFSE at a final concentration of 25 μM for 30 minutes at 37°C in the dark. Specifically, 2.5 μL 5 mM CFSE was added to 497.5 μL ECVs. After 30 minutes of labeling, excess CFSE was washed out by spinning the ECVs in a large volume of HBSS for 70 minutes at 100,000 × g. After this wash step, CFSE-labeled ECVs were resuspended in a volume 1/500th the original starting volume of conditioned media.

### Immunoblotting

Western blotting was performed on B16-GFP-Cre ECVs using a Cre-specific primary antibody. ECVs were lysed by boiling in Laemmli loading buffer for 5 minutes in reducing conditions. Purified Cre recombinase (New England Biolabs, Ipswich, MA, USA) was used as a positive control. Samples were resolved using SDS-PAGE, transferred onto a PVDF membrane, and imaged using the ECL method. Antibodies against the following epitopes were used: Cre (Cell Signaling, Danvers, MA, USA), rabbit IgG (Santa Cruz Biotechnology, Dallas, TX, USA).

### Cre PCR

Total RNA was extracted from purified ECVs or cells using the Trizol reagent (Life Technologies, Carlsbad, CA, USA) and then subjected to cDNA synthesis using the High Capacity cDNA Reverse Transcriptase kit (Applied Biosystems, Foster City, CA, USA) according to the manufacturer’s protocol. A ND100 spectrophotometer (Nanodrop) was used to assess the concentration and purity of RNA prior to cDNA synthesis. PCR was performed on cDNA using Cre-specific primers (see Supplementary Table 1) that amplify a 729 bp-sized fragment under the following thermal cycle conditions: 10 minutes at 94°C, followed by thirty cycles consisting of: 45 seconds at 94°C, 45 seconds at 62°C, and 45 seconds at 72°C. PCR reactions were concluded with incubation for 10 minutes at 72°C. Primers specific for *Gapdh* were used as control. After completion, the PCR reactions were loaded on to a 1% TAE agarose gel, electrophoresed, and imaged with ethidium bromide (Sigma, St. Louis, MO, USA).

To probe for Cre DNA in tdTomato^+^ clonal cell lines, total genomic DNA was isolated from each cell line using the ethanol precipitation method and assayed for concentration and purity using a ND100 spectrophotometer (Nanodrop). DNA was subjected to PCR using Cre-specific primers and the following thermal conditions: [45 seconds at 94C; 45 seconds at 62C; 45 seconds at 72C] repeated thirty times. After PCR was completed, each PCR reaction was loaded on to a 1% agarose gel, electrophoresed, and imaged with ethidium bromide (Sigma, St. Louis, MO, USA).

### Imaging

For live cell imaging of *in vitro* Cre transfer, B16-GFP-Cre cells were added to adherent reporter MEF that had been labeled with 5 uM of CellTracker Blue (Molecular Probes #C2110) according to the manufacturer’s protocol. The video recording was initiated after 2 hours. Images were collected every 3–4 minutes with xyzt acquisition mode using an Axio Observer.Z1 microscope with the LSM 700 scanning module (Zeiss, Jena, Germany). Cultures were maintained at 37°C, 5% CO_2_ using a Heating Insert P Lab-Tek S1 with an Incubator PM S1 (Zeiss, Jena, Germany).

For imaging of *in vivo* Cre transfer, B16-GFP-Cre tumors were grown in reporter mice as described above. After 18–20 days, mice were sacrificed, and tumors were harvested, coated in OCT, and flash frozen in liquid nitrogen. Cryosectioning was then performed to generate tumor sections 15 μm thick, which were then imaged with a Nikon D-Eclipse C1TE2000 confocal microscope (Nikon, Tokyo, Japan).

### DNA content analysis by flow cytometry

DNA content of B16-GFP-Cre and tdTomato^+^ clonal cell lines was measured using flow cytometry as previously described [63]. 1 × 10^6^ cells were resuspended in 0.5 ml HBSS, to which was added 4.5 ml 70% ethanol (dropwise). After incubation for 60 minutes at 4^°^C, the cells were washed three times with HBSS and incubated in the presence of 7-AAD (2 μg/ml) for 20 minutes at 4^°^C. Finally, the cells were washed, resuspended in FACS staining buffer, and analyzed in the PerCP channel using linear voltage setting. These analyses reveal two peaks that represent cells in the G1 (left peak) or G2 (right) phase of the cell cycle. The MFI of the G1 peak was determined for each cell line, and the relative ploidy was calculated by normalizing the G1 MFI of each cell line against MEF, which was set at a ploidy of “2n”.

### Karyotype analysis

For karyotype analysis, adherent cells were treated with 0.1 μg/ml of KaryoMAX Colcemid Solution (Life Technologies, Carlsbad, CA, USA) for 4 hours. Cells were then harvested, treated with a hypotonic solution of 0.8% sodium citrate for 10 mins at room temperature, washed, treated with Carnoy’s fixative (75% MeOH, 25% glacial acetic acid) for 10 minutes at room temperature, and washed again. These four steps (hypotonic solution, wash, fix, wash) were repeated two additional times. After fixation, a drop of cells in fixative was released onto a slide and allowed to sit until dry. The slide was then stained with Giemsa for 20 minutes and mounted in mounting medium for analysis. At least 15 cell-karyotypes were counted for each cell line.

### Quantitative RT-PCR

RNA was extracted from cell lines using TRIzol reagent (Life Technologies, Carlsbad, CA, USA) and measured with a ND100 spectrophotometer (Nanodrop) for concentration and purity. RNA was then subjected to cDNA synthesis using High Capacity cDNA Reverse Transcription Kit (Applied Biosystem, Foster City, CA, USA) according to the manufacturer’s protocol. qPCR was performed using SYBR Green PCR Master Mix (Applied Biosystem, Foster City, CA, USA) and the following thermal cycle conditions: 10 minutes at 95°C, followed by forty cycles consisting of: 10 seconds at 95°C, 60 seconds at 60°C using a CFX96 Touch Real-Time PCR Detection System (Bio-Rad Laboratories, Irvine, CA, USA). Gene expression was analyzed with the 2^−ΔΔCt^ method normalized against *Hprt*. Primer sequences are listed in Supplementary Table 1.

### *In vitro* chemoresistance assays

To measure chemoresistance of tdTomato^+^ cells from *in vitro* co-cultures, B16-GFP-Cre cells were co-cultured with reporter MEF or BMDM for 24 hrs at a ratio of 1:10 and then treated with 0.1 or 10 μM paclitaxel (TEVA Pharmaceuticals, Petah Tikva, Isreal) or vehicle control for 24 hrs. Then the frequency of tdTomato^+^ cells in each co-culture was measured using FACS.

To measure chemoresistance of tdTomato^+^ and B16-GFP-Cre clonal cell lines, each cell line was seeded in a 24 well plate, grown for 24 hrs, and then treated with 10 μM paclitaxel, 10 μM doxorubicin (Bedford Laboratories, Bedford, OH, USA), or vehicle control for 24 hours. Each condition was performed in triplicate. Then cells were harvested and counted, and the relative survival of each cell line in the presence of each drug was calculated by dividing the number of cells in the drug treated condition by the number of cells in the control condition. Viability was determined based on the exclusion of 7-AAD as measured by flow cytometry.

### *In vivo* chemoresistance assay

To determine if *in vivo*-derived tdTomato^+^ cells exhibited increased resistance to chemotherapy, 1 × 10^6^ B16-GFP-Cre cells were injected into reporter mice that were then treated with a chemotherapy regime that shrunk tumors by approximately 50%. The chemotherapy regimen consisted of intraperitoneal injections of paclitaxel (15 mg/kg) or vehicle control on days 6, 8, 10, 12, 14, and 16 post-tumor cell injection. Once tumors reached 10 × 10 mm, mice were sacrificed and tumors were harvested/ prepared into a single cell suspension and analyzed by FACS to calculate the frequency of tdTomato^+^ cells.

### Quantification and statistical analysis

GraphPad Prism 7 (GraphPad Software, La Jolla, CA, USA) was used to analyze all datasets. Pairwise comparisons were generated with two-tailed *t* tests. Variance between groups was calculated with the *F* test using a confidence level of α = 0.01. Definitions of center/ dispersion measurements and *n* values are all indicated in the associated figure legends for each figure. P-values are represented as follows: ^*^p < 0.05, ^**^p < 0.01, ^***^p < 0.001, ^****^p < 0.0001.

## SUPPLEMENTARY MATERIALS FIGURES, TABLE AND VIDEOS












