# Supplementary material for: Cell-cell fusion as a mechanism of DNA exchange in cancer
Source: Oncotarget. 2017 Dec 27;9(5):6156–73. doi: 10.18632/oncotarget.23715 (PMC5814202; doi:10.18632/oncotarget.23715)
Supplement: Supplementary file 1 [file oncotarget-09-6156-s001.pdf]

Cell-cell fusion as a mechanism of DNA exchange in cancer

SUPPLEMENTARY MATERIALS

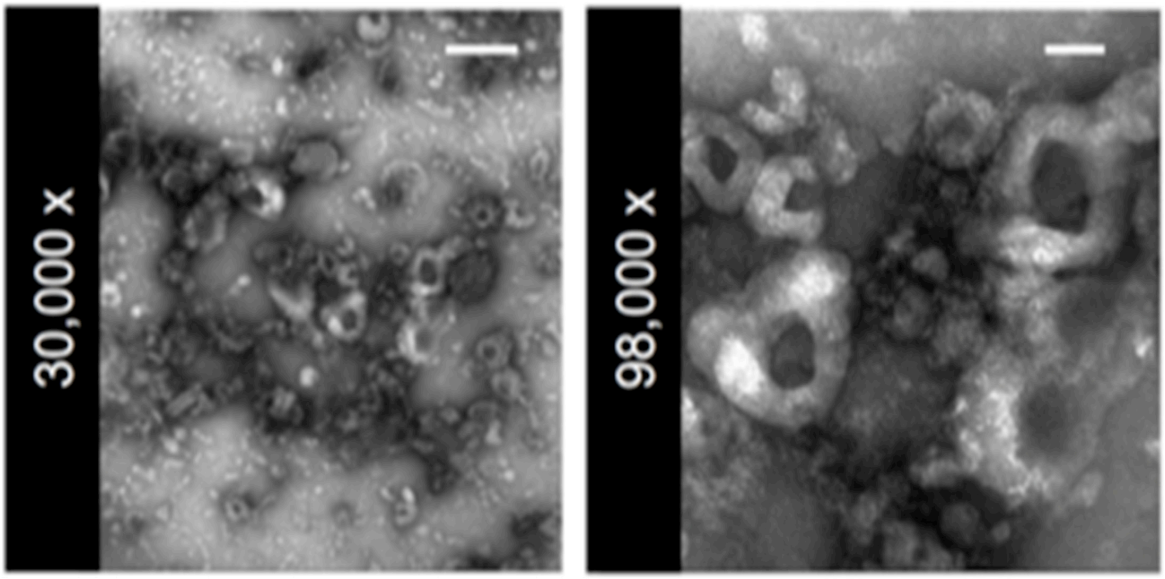

Supplementary Figure 1: Size and morphology of B16-GFP-Cre ECVs. Electron micrographs showing size and morphology of vesicles isolated from the conditioned media of B16-GFP-Cre cells via differential ultracentrifugation. Size bar equals 200 nm (30,000×) or 50 nm (98,000×).

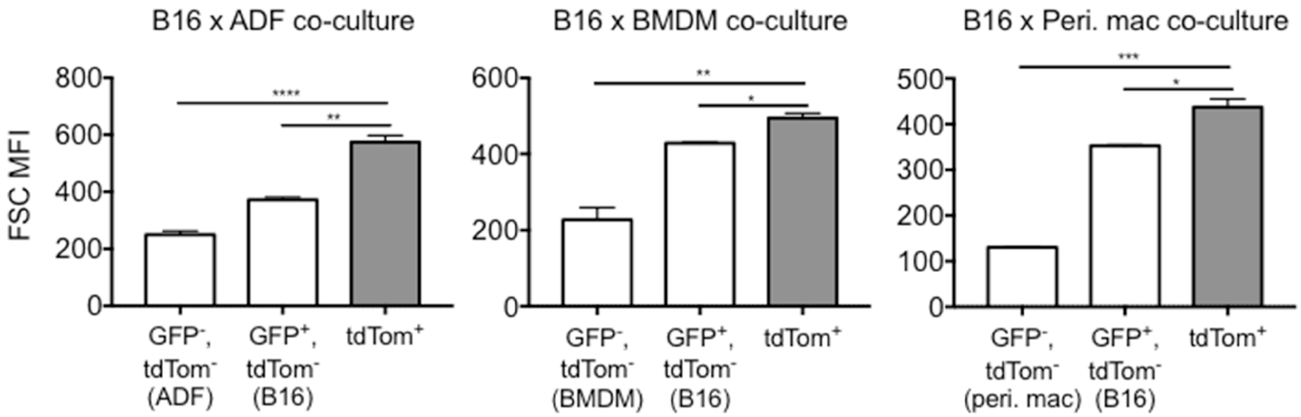

Supplementary Figure 2: (Related to Figure 3B): Reporter⁺ cells are larger than B16 cells and Reporter⁻ cells. FSC analysis of three populations of cells (GFP–/tdTomato–, GFP+/tdTomato–, tdTomato+) from 48 hr co-cultures of B16-GFP-Cre cells with various reporter cells (indicated).

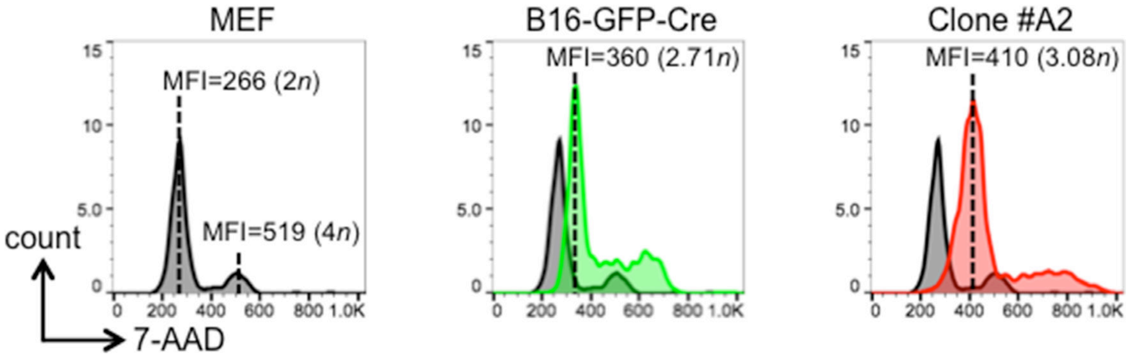

Supplementary Figure 3: (Related to Figure 4D): FACS-based quantification of DNA content. Representative histograms showing DNA content of MEF, B16-GFP-Cre, and one of the twenty tdTomato+ clones (#A2). The two peaks in each histogram correspond to cells in the G1 (left peak) or G2 (right peak) phase of the cell cycle. For B16-GFP-Cre and each of the twenty tdTomato+ clones, relative ploidy was calculated by normalizing the MFI of the G1 peak to MEF, which were set as 2*n*.

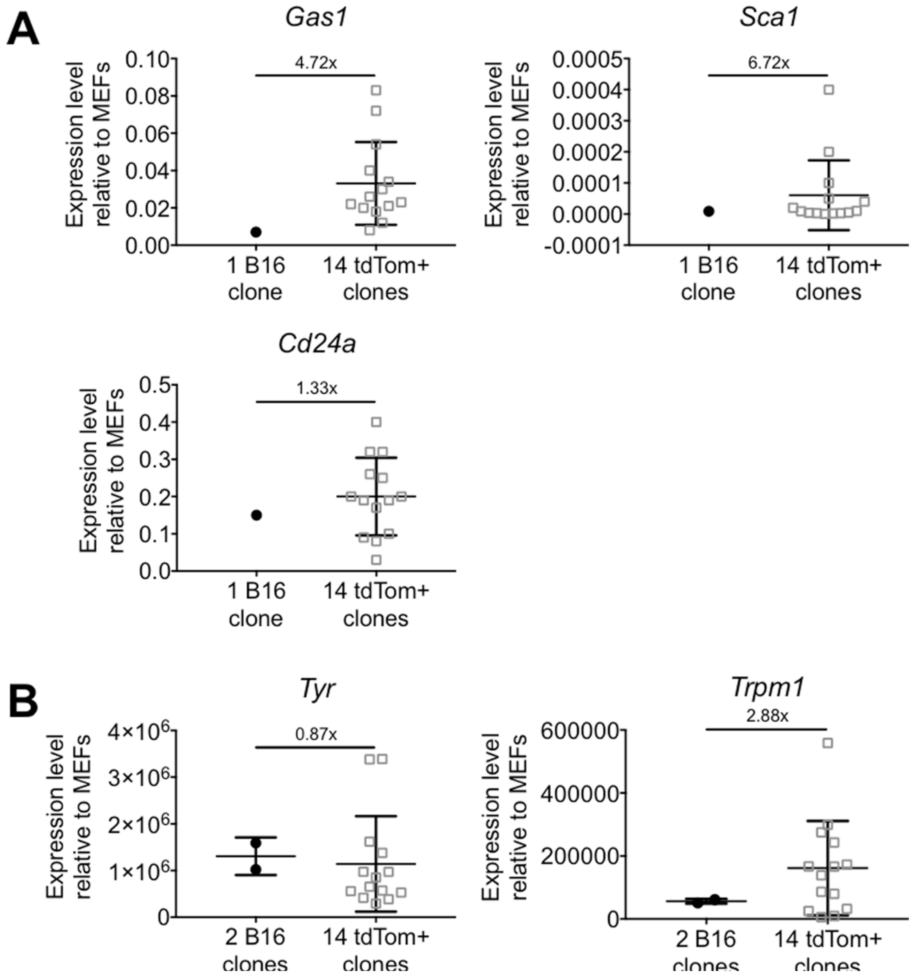

Supplementary Figure 4: (Related to Figure 5C): B16xMEF hybrids express both B16- and MEF-restricted genes. (**A**) Comparison of the average expression level of three candidate "MEF genes" in B16-GFP-Cre cells and fourteen tdTomato+ clones (n = 2 independent experiments per data point). For the tdTomato+ clones, data is represented as mean +/− SEM. (**B**) Comparison of the average expression level of two candidate "B16 genes" in two B16-GFP-Cre clones and fourteen tdTomato+ clones (n = 2 independent experiments per data point). Data is represented as mean +/− SEM.

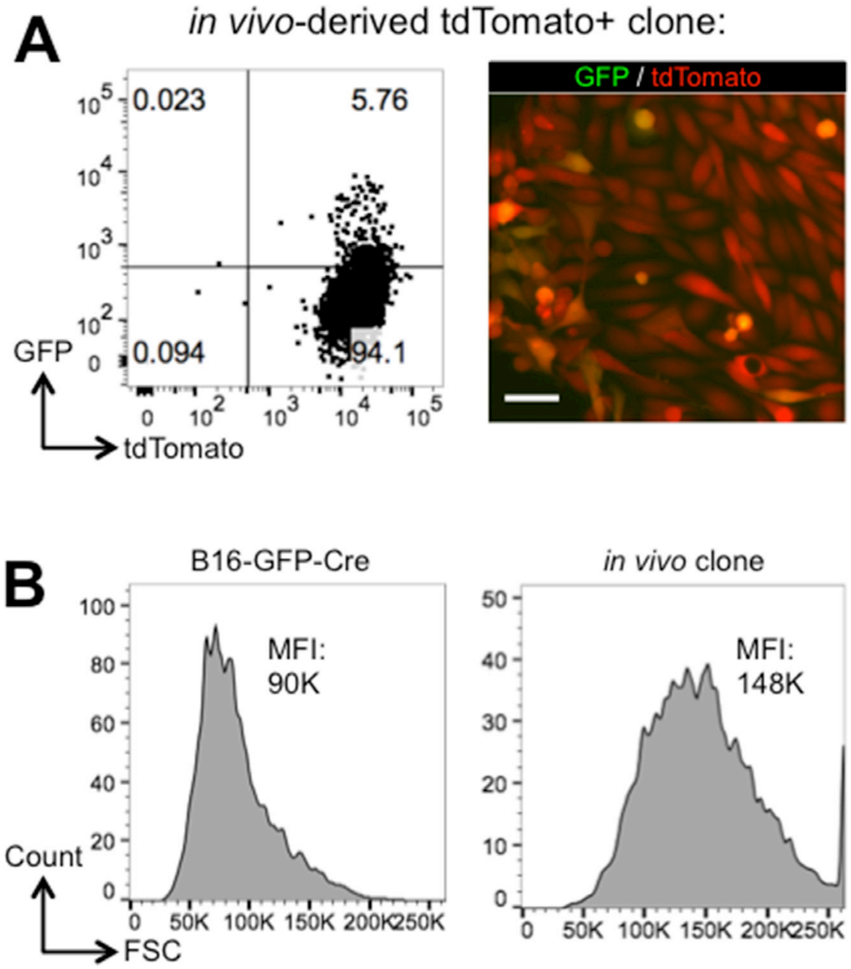

Supplementary Figure 5: (Related to Figure 7E): Cell-cell fusion occurs *in vivo.* (**A**) Representative FACS plot and fluorescent micrograph showing GFP and tdTomato expression in the *in vivo*-derived tdTomato$^+$ clone after four passages. Scale bar = 100 μM. (**B**) Representative histograms showing FSC (as a read-out for cell size) of B16-GFP-Cre and the *in vivo*-derived clone.

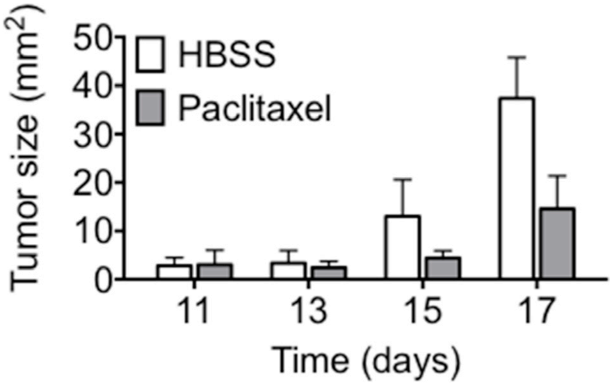

Supplementary Figure 6: (Related to Figure 7F): Paclitaxel treatment shrinks B16-GFP-Cre tumors *in vivo.* (**A**) Bar graph showing the size of B16-GFP-Cre tumors that were grown in mice treated with paclitaxel or HBSS (control). Data are represented as mean +/– SEM (*n* = 6 mice per group).

Supplementary Video 1: (Related to Figure 3): Cell-cell fusion mediates the transfer of Cre from B16 melanoma cells to MEF. (**A**) Live-cell imaging of B16-GFP-Cre cells co-cultured with CellTracker Blue-labeled reporter MEF between 2 and 18 hours. Scale bar equals 50 μM. Green represents GFP, blue represents CellTracker Blue, and red represents tdTomato. See Supplementary_Video_1

Supplementary Video 2: (Related to Figure 3): Cell-cell fusion mediates the transfer of Cre from B16 melanoma cells to MEF. (**A**) Live-cell imaging of B16-GFP-Cre cells co-cultured with CellTracker Blue-labeled reporter MEF between 2 and 18 hours. Scale bar equals 50 μM. Green represents GFP, blue represents CellTracker Blue, and red represents tdTomato. See Supplementary_Video_2

Supplementary Video 3: (Related to Figure 3): Cell-cell fusion mediates the transfer of Cre from B16 melanoma cells to MEF. (**A**) Live-cell imaging of B16-GFP-Cre cells co-cultured with CellTracker Blue-labeled reporter MEF between 2 and 18 hours. Scale bar equals 50 μM. Green represents GFP, blue represents CellTracker Blue, and red represents tdTomato. See Supplementary_Video_3

Supplementary Video 4: (Related to Figure 3): Reporter^{+} cell undergoing apoptosis. (**A**) Live-cell imaging of B16-GFP-Cre cells co-cultured with CellTracker Blue-labeled reporter MEF between 18 and 40 hours. Scale bar equals 50 μM. Green represents GFP, blue represents CellTracker Blue, and red represents tdTomato. See Supplementary_Video_4

Supplementary Video 5: (Related to Figure 3): Cell-cell fusion mediates the transfer of Cre from B16 melanoma cells to MEF. (**A**) Live-cell imaging of B16-GFP-Cre cells co-cultured with unlabeled reporter MEF between 2 and 20 hours. Scale bar equals 50 μM. Green represents GFP and red represents tdTomato. See Supplementary_Video_5

Supplementary Table 1: Primer sequences

**Table of primers**	
**Name**	**Sequence (5′ to 3′)**
Bmp4 (forward)	GGCTGGCCATTGAGGTGACT
Bmp4 (reverse)	TCGGCTGATTCTGACATGCTG
Cd24a (forward)	CTGCTGGCACTGCTCCTAC
Cd24a (reverse)	GGTGGTGGCATTAGTTGGAT
CRE (forward)	GCCAGCTAAACATGCTTCATC
CRE (reverse)	ATTGCCCCTGTTTCACTATCC
Fgf2 (forward)	TCCAGTTGGTATGTGGCACTGA
Fgf2 (reverse)	CAGTATGGCCTTCTGTCCAGGTC
Gapdh (forward)	GGAGCCAAAAGGGTCATCAT
Gapdh (reverse)	GTGATGGCATGGACTGTGGT
Gas1 (forward)	CCCTCTTCTGTGCGGTTTTC
Gas1 (reverse)	CACCGTTCAGCACTCTGAGTC
Hprt (forward)	GCTTGCTGGTGAAAAGGACCTCTCGAAG
Hprt (reverse)	CCCTGAAGTACTCATTATAGTCAAGGGCAT
Met (forward)	GCATGTCAGCATCGCTCAA
Met (reverse)	TGCAGGCCCAGCTGTTTC
Mitf (forward)	GCCTTGTTTATGGTGCCTTC
Mitf (reverse)	GTCCTCCTCCCTCTACTTTCTGT
Sca1 (forward)	CTCTGAGGATGGACACTTCT
Sca1 (reverse)	GGTCTGCAGGAGGACTGAGC
Trpm1 (forward)	GAGCTGAAGGAGGCTAGGCTG
Trpm1 (reverse)	CTTGGTGTCCTCTCCTGTTGT
Tyr (forward)	CCTCCTGGCAGATCATTTGT
Tyr (reverse)	ATCGCATAAAACCTGATGGC
